# Metabolic disruption by mycotoxins: focus on metabolic endpoints steatosis, adipogenesis and glucose metabolism in vivo and in vitro

**DOI:** 10.1007/s00204-025-03957-w

**Published:** 2025-02-09

**Authors:** Tia Heikkinen, Jenni Küblbeck, Jaana Rysä

**Affiliations:** 1 Faculty of Health Sciences, School of Pharmacy, University of Eastern Finland, P.O. Box 1627, 70211 Kuopio, Finland; 2 A.I. Virtanen Institute for Molecular Sciences, University of Eastern Finland, Kuopio, Finland

**Keywords:** Cholesterol, Glucose tolerance, Insulin tolerance, Lipid accumulation, Metabolic disrupting chemicals

## Abstract

Metabolic disruption encompasses the processes leading to adverse effects to major metabolic organs, such as liver and pancreas after exposure to e.g., environmental chemicals. As some mycotoxins act as endocrine disruptors, their structural similarity may lead to effects in lipid and glucose metabolism as well. Via systematic literature search, we mapped the potential of mycotoxins to cause metabolic disruption. Our systematic data search involved mycotoxin keywords combined with metabolic disruption keywords. The retrieved 31 studies revealed 24 in vivo studies, and 18 in vitro studies in total of 13 different mycotoxins. Most studied parameters were triglycerides from blood or liver, followed by total cholesterol and glucose or insulin levels. In vitro studies most often aimed to reveal mechanisms of metabolic disruption, but common metabolic parameters (lipid or cholesterol accumulation). In general, mycotoxin exposure showed a trend towards positive metabolic effects, such as reduction of blood triglycerides levels. Emodin was the most studied mycotoxin. Other mycotoxins were studied in one to three studies. Positive effects were also identified for equisetin, fumonisin B1, fumigaclavine C and ergostatrien-3-B-ol. Adverse effects (e.g. increased lipid deposition to liver) were identified for aflatoxin B1, ochratoxin A, deoxynivalenol, citreoviridin, T-2 toxin and paxilline. As demonstrated by the evaluated in vivo and in vitro studies, mycotoxins seem to have more positive than negative effects on metabolism. However, based on the available data, a general conclusion on the role of mycotoxins as a group cannot be made.

## Introduction

Obesity, diabetes, and other metabolic diseases have reached epidemic proportions and constitute a major public health concern worldwide (Noubiap et al. 2022; Artasensi et al. 2023). Increased caloric intake and sedentary lifestyle are well-accepted major risk factors for metabolic disorders (Wu et al. 2022; Fahed et al. 2022), but also environmental factors have negative impact on metabolic health. A hypothesis concerning the link between obesity and environmental chemicals apart from overeating and inactivity was proposed already over 20 years ago (Baillie-Hamilton 2002) and currently the link between metabolic disorders and endocrine disrupting chemicals (EDCs) is well-established (Haverinen et al. 2021; Heindel et al. 2022). EDCs are a group of heterogenous compounds that can have multiple adverse effects on the endocrine system including reproductive health and thyroid disorders. A subset of endocrine disruptors have been proposed to act as metabolic disrupters as well, and the possible mechanisms of these metabolism disrupting chemicals (MDCs) are numerous—epigenetic alterations, disruption in nuclear receptor (NR) homeostasis as well as mitochondria disrupting effects have been studied (Heindel et al. 2017, 2022).

Mycotoxins are naturally produced toxic compounds by certain types of fungi. A proposed definition of mycotoxins incorporates two levels: classification as secondary metabolite of microfungi, and exerting “toxic activity” in the effectiveness level of 50% below 1 mM of exposure in vitro (Taevernier et al. 2016), reflecting a potential for much more mycotoxins to be discovered in the future. The definition covers only human or animal vertebrate cell models. The occurrence and frequency of mycotoxins is presumed to shift favoring thermotolerant species due to climate change (Zingales et al. 2022). Different mycotoxins have variable structures as well as adverse effects (e.g. Alexander et al. 2011; Benford et al. 2014; Schrenk et al. 2020a, b). Existing evidence supports the fact that certain mycotoxins act as EDCs (Alexander et al. 2011; Demaegdt et al. 2016; Balló et al. 2023). For example, zearalenone has been classified as EDC by the European Food Safety Authority (EFSA) (Alexander et al. 2011), and aflatoxin B1 has been shown to have EDC potential (Kościelecka et al. 2023).

Most well-characterized mycotoxins (e.g. aflatoxins, ochratoxin A, zearalenone) have been assessed by EFSA risk assessment working groups regarding metabolic disruption properties (Alexander et al. 2011; Schrenk et al. 2020a, b). Mostly in vitro based studies show that mycotoxins have a potential to act as obesogens, as demonstrated by Demaegdt et al. (2016) where all 13 studied mycotoxins or their metabolites acted as peroxisome proliferator-activated receptor (PPAR)-γ2 antagonists. Metabolic disruption by mycotoxin has been reported also in vivo, such as an increase in hepatic triglyceride accumulation after citreoviridin and deoxynivalenol exposure in mice (Feng et al. 2017; Barbouche et al. 2020). However, the role of mycotoxins as metabolic disruptors is unclear.

In this study, we systematically searched literature to screen whether mycotoxins possess metabolic disruption potential. According to our knowledge, a systematic characterization of the metabolic disruption capacity of mycotoxins has not been previously conducted. The aim of this study is to review the current state of research and to reveal whether mycotoxins could be characterized as metabolic disruptors.

## Materials and methods

### Search strategy

The systematic literature search was conducted on 3.11.2023 (articles from 01.01.1900–03.11.2023) from two independent databases (PubMed and Web of Science) in accordance with the Preferred Reporting Items for Systematic Reviews and Meta-Analyses (PRISMA) (Page et al. 2021). In short, an initial list of keywords was adopted from EFSA’s scientific report on mycotoxin mixtures (Battilani et al. 2020). Additional terms (e.g. gliotoxin) were added and semisynthetic derivatives removed to ensure that the search strategy included all relevant search terms that were of interest to the study. The search strategy was developed with the assistance of a librarian and included search terms related to adverse metabolic endpoints and endocrine disruption. Further studies were identified in reviewed articles. The detailed search strategy and keywords are listed in supplementary information.

### Data collection and extraction

Articles were managed with Covidence systematic review software (Veritas Health Innovation, Melbourne, Australia; available at www.covidence.org). After removal of duplicates, the title and abstract of each article were screened by two independent reviewers, (T.H. and J.R.) and discrepancies were resolved by a third reviewer (J.K.). All included articles then underwent full-text review conducted by two independent study reviewers (T.H. and J.R.), with a third reviewer (J.K.) settling discrepancies. The data was extracted by all reviewers.

The medical subject heading terms (MESH) used in PubMed retrieved many additional keywords, that were not useful regarding this study. Thus, all the irrelevant findings were left out in the screening phase. Table 1 provides a list of inclusion and exclusion criteria in the screening phase of the study. Finally, all reviewers assessed the effect of the mycotoxins on glucose and lipid metabolism related endpoints (increase, decrease or no change).

**Table 1:** Inclusion and exclusion criteria of the study

Inclusion criteria	Exclusion criteria
In vivo studies in mammalian species	Drugs or derivatives of mycotoxins (e.g. bromocriptine)
In vitro studies with relevant cell models (e.g. hepatic and pancreatic cells, adipocytes)	No metabolic endpoint (e.g. glucose or lipid metabolism)
Exposure to one single mycotoxin in the experimental setting	Mechanistic studies (e.g. receptor binding, RNA expression) without relevant metabolic endpoint
Endpoint related to metabolic disruption (e.g. altered lipid accumulation in cells or tissue)	Non-relevant cell models (e.g. intestinal cell lines)
Experimental setting allowed identification of the relevant endpoint	Article not in English

## Results

### Study selection

**Fig. 1:**
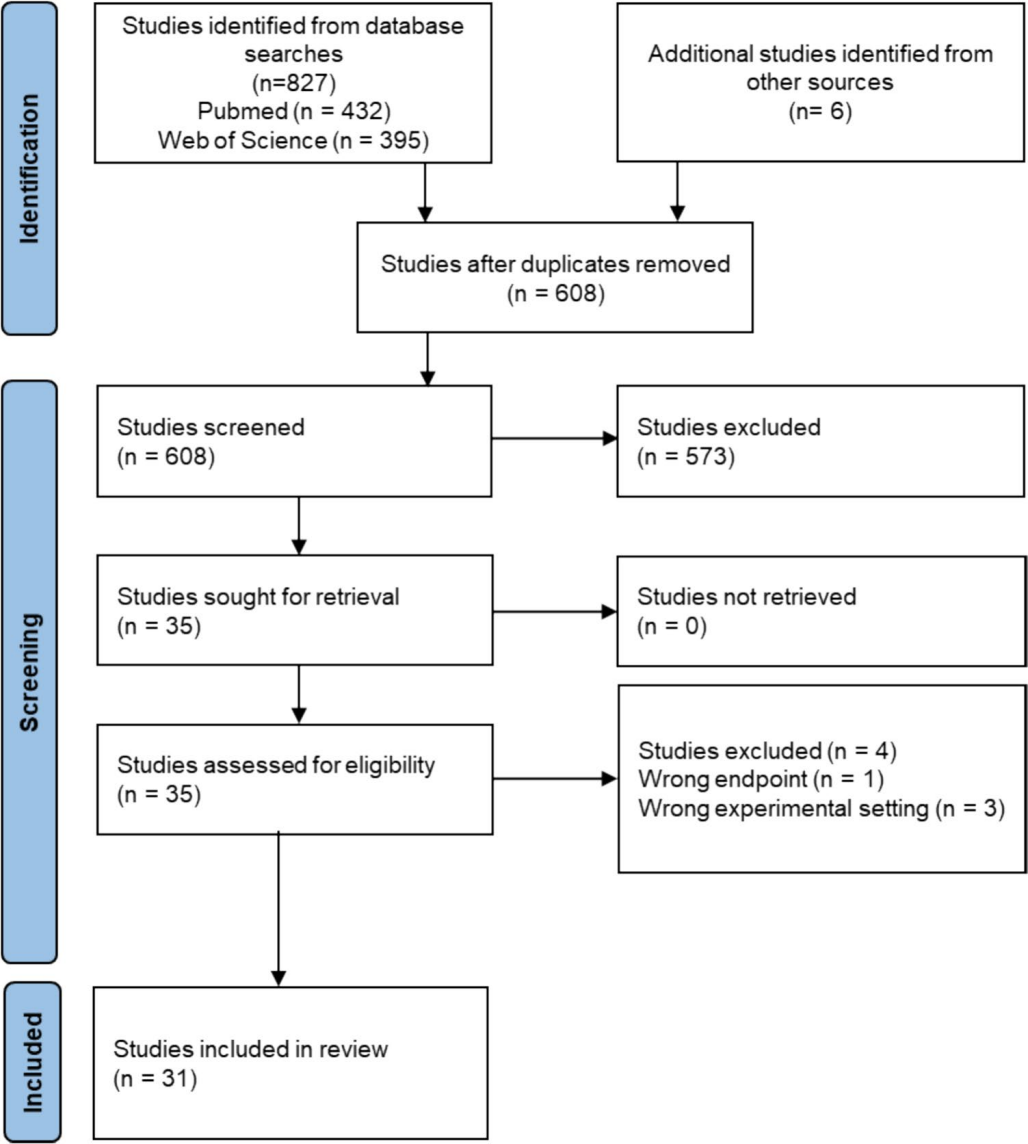
PRISMA flow diagram indicating the selection process of the articles in the present systematic literature search

Pubmed retrieved 432 articles and Web of science retrieved 395 articles with the defined keywords. Of 608 articles eligible for title and abstract screening, 35 were eligible for full text screening and 31 articles were included in the review (Fig. 1). The search included 73 different mycotoxins and studies on metabolic disruption were identified for 13 mycotoxins. Out of 31 studies in total, 24 (77%) included in vivo work and 18 (58%) included in vitro studies. In 12 (39%) studies, both in vivo and in vitro methods were used.

### Metabolic effects of mycotoxins in vivo

Emodin was the most studied mycotoxin in vivo, demonstrated by 10 studies (42% of in vivo studies) (Table 2). In addition, deoxynivalenol and aflatoxin B1 were studied in three studies, and fumigaclavine C, fumonisins and T2 toxin in two studies of the total 24 studies. There were single studies on citreoviridin, equisetin, ochratoxin A and ergostatrien-3-B-ol. The most commonly used animal was C57BL/6J mouse (13/24; 54%), followed by Sprague–Dawley rats (4/24 studies; 17%), and ICR mice (2/24; 8%). In addition, apolipoprotein E-deficient (ApoE^−/−^) mice, leptin deficient *ob/ob* mice, Wistar rats and pigs were used in one study. The length of studies ranged from acute (3–24 h) exposure to 3 months, with most common lengths being around 3–4 weeks of exposure. The doses varied from 0.005–80 mg/kg in mice and 40–160 mg/kg in rats. In several studies, exposure to mycotoxin was combined with obesogenic diet (10 studies in mice, 3 studies in rats).

**Table 2:** In vivo studies on associations of mycotoxins and lipid and glucose metabolism

Mycotoxin	Species/strain (sex)	Duration	Dosage	Group size	Diet^a^	Outcome	Lipid	Glucose	References
Aflatoxin B1	C57BL/6 mice	3 weeks	2.5 mg/kg bw/d every other day	n = 6	NM	Increased liver steatosis Increased liver and serum TG and TC	↑	n.a	Che et al. (2023)
	C57BL/6 J mice (male)	1 month	0.6 mg/kg bw/d once in two days	n = 5	NM	Increased lipid accumulation in liver Increased liver TG, TC	↑	n.a	Ren et al. (2020)
Citreoviridin	ICR mice (male)	6 weeks	0.1–0.3 mg/kg bw/d	n = 6	NM	Increased accumulation of lipids and TG in liver No change in serum TC, LDL-C and HDL-C Increased serum TC/HDL-C ratio	↑	n.a	Feng et al. (2017)
Deoxynivalenol	C57BL/6 J mice (male)	3–24 h	12.5 mg/kg bw/d	n = 5–14	Standard	Increased lipid accumulation and liver steatosis No change in hepatic glycogen content Increased plasma TG Decreased fasting blood glucose	↑	↓	Barbouche et al. (2020)
	B6C3F1 mice (female)	148 days	10 mg/kg bw/d	n = 8	HFD (45/60% fat)	No change in plasma glucose levels Decreased plasma insulin and leptin levels	n.a	↓	Kobayashi-Hattori et al. (2011)
	Pigs	28 days	4 mg/kg bw/d	n = 7	Standard	Decrease in serum cholesterol, TG and BAs	↑	n.a	Zong et al. (2022)
Emodin	SD rats (male)	10 weeks	40 mg/kg bw/d	n = 8	Standard (5% fat)	No change in lipid metabolism	↔	↔	Alisi et al. (2012)
	SD rats (male)	10 weeks	40 mg/kg bw/d		HFD/HF (58% fat)	Decreased liver steatosis and inflammation Decreased plasma TG No change in plasma TC Decrease in fasting blood glucose, insulin and HOMA-IR	↓	↓	Alisi et al. (2012)
	SD rats	3 last weeks of 8-week HFD	50 mg/kg bw/d twice per week	n = 8	HFD (60% fat)	Decreased size of adipocytes in groin but not in epididymis No change in liver histology Decreased plasma TC Reduced TG in liver and epididymis	↓	n.a	Fang et al. (2022)
	C57BL/6 J mice (male)	35 days	50/100 mg/kg bw/d twice per day	n = 8	HFD (60% fat)	At dose of 100 mg/kg bw/d: Reduced mesenteric, perirenal and subcutaneous fat Decreased serum TG and TC levels Decreased serum glucose and insulin	↓	↓	Feng et al. (2010)
	C57BL/6 J mice (male)	6 weeks	40 or 80 mg/kg bw/d	n = 8	HFD (60% fat)	At dose of 80 mg/kg bw/day: Decreased TC, TG and lipid deposition in liver Decreased epididymal fat weight and adipocyte diameter in BAT and WAT Decreased blood TG, LDL-C, TC and glucose No change in blood HLD-C and insulin	↓	↓	Li et al. (2016)
	C57BL/6 J mice (male)	8 weeks	20/40/80 mg/kg bw/d	n = 10	HFD (standard diet added with 15% fat, 1% cholesterol)	At dose of 80 mg/kg bw/d: Improved HFD-induced lipid accumulation in liver Decreased serum TG and TC Reduced fasting blood glucose and insulin Improved glucose tolerance and insulin sensitivity	↓	↓	Shen et al. (2021)
	Wistar rats (male)	8 weeks	20/40/80 mg/kg bw/d	n = 8	Standard (11 kcal% fat, followed by HFD (45% fat)	Decreased bw and eWAT, pWAT, mWAT and iWAT Decreased plasma TG, TC, LDL-C and FFAs Decreased plasma glucose and insulin	↓	↓	Tzeng et al. (2012)
	*ob/ob* mice	26 days	25/50 mg/kg bw/d twice per day	n = 6–8	NM	At dose of 50 mg/kg bw/d: Decreased mesenteric fat Decreased serum TG No change in serum TC Decreased non-fasting and fasting blood glucose levels. Improved glucose tolerance	↔	↓	Wang et al. (2012)
	SD rats (male)	8 weeks	40/80/160 mg/kg bw/d	n = 10	HFD (25% fat, 2% cholesterol)	At dose of 160 mg/kg bw/d: Decreased liver index and hepatic and serum TG and TC Decreased serum TG, TC, and LDL-C	↓	n.a	Wang et al. (2017)
	C57BL/6 J mice (male)	3 weeks	1.5 mg/kg bw/d	n = 8–15	HFD^b^ with streptozotocin	Decreased serum TG and TC Increased serum HDL-C No change in serum LDL-C Decreased serum glucose and improved glucose and insulin tolerance	↓	↓	Xue et al. (2010)
	C57BL/6 J mice (male)	3 months	80 mg/kg bw/d	n = 5–8	Standard	No change in adipocyte number No change in fasting serum TC, LDL-C and HDL-C Decreased fasting serum glucose No change in fasting serum insulin	↔	↔	Yu et al. (2021)
	C57BL/6 J mice (male)	3 months	80 mg/kg bw/d	n = 5–8	HFD (60% fat)	Reduced adipocyte size and number, rWAT, eWAT and accumulation of lipids in adipocytes Decreased serum TC, HDL-C and LDL-C No change in serum TG Decreased serum glucose and insulin levels Improved insulin sensitivity	↓	↓	Yu et al. (2021)
Equisetin	C57BL/6 J mice (male)	6 weeks	20/40/80 mg/kg bw/d	n = 3–8	HFD (60% fat)	Reduced fat tissue weights Decreased size of adipocytes Reduced blood TG, TC and LDL-C No change in blood HDL-C	↓	n.a	Xu et al. (2022)
Ergostatrien-3-B-ol	C57BL/6 J mice (male)	4 weeks	10/20/40 mg/kg bw/d once per day	n = 9	HFD (45% fat)	In all exposure groups: Decrease in retroperitoneal epididymal and visceral fat Decrease in TC, TG, HDL-C and FFAs Reduced blood glucose levels and Hba1c At dose of 40 mg/kg bw/d: Decrease in mesenteric fat and BAT Reduced hypertrophy of adipose tissue Decreased ballooning degeneration of hepatocytes Reduced blood LDL-C and leptin Decrease in blood insulin	↓	↓	Kuo et al. (2015)
Fumigaclavine C	C57BL/6 mice (male)	4 weeks (from 16 weeks of age)	10 mg/kg bw/d	n = 8–10	HFHC (20% fat, 0.15% cholesterol)	No change in lipid parameters	↔	n.a	Du et al. (2014)
	ApoE^−/−^ mice	4 weeks (from 16 weeks of age)	5/10/20 mg/kg bw/d	n = 8–10	HFHC (20% fat, 0.15% cholesterol)	Reduced lipid accumulation in liver Decrease in serum TG, TC and LDL-C No change in serum HDL-C	↓	n.a	Du et al. (2014)
	C57BL/6 J mice (male)	10 weeks	10/20/40 mg/kg bw/d, 3 times a week	n = 10	HFD (50% fat)	Reduced lipid accumulation in liver Reduced serum TC, TG, LDL-C and FFAs No change in serum HDL-C	↓	n.a	Yu et al. (2023a)
Fumonisin B1	C57BL/6 J mice (male)	3 weeks (from 12 weeks to16 weeks)	10 mg/kg bw/d	n = 6	Standard	No change in liver morphology or plasma lipid and glucose parameters	↔	↔	Dopavogui et al. (2023)
	C57BL/6 J mice (male)	3 weeks (from 12 weeks to16 weeks)	10 mg/kg bw/d	n = 6	HFD (60% fat)	Reduced liver steatosis and TG Reduced fasting blood glucose levels Increased liver inflammation	↓	↓	Dopavogui et al. (2023)
Ochratoxin A	C57BL/6 mice (male)	12 weeks	1 mg/kg bw/d	n = 12	Standard	Increase in liver steatosis and TG No change in serum TG	↑	n.a	Zheng et al. (2021)
T2 toxin	ICR pregnant mice (female)	28 days, from late gestation to lactation	0.005–0.05 mg/kg bw/d	n = 6	NM	Postnatal changes: Increase in lipid accumulation in the liver Changes towards increase in liver TC, TG and LDL-C No change in liver HDL-C Increase in serum TG No change in serum TC and LDL-C Decrease in serum HDL-C Increase in blood glucose	↑	↑	Kang et al. (2020)
	C57BL/6 J mice (male)	9 weeks	1 mg/kg bw/d, 3 times a week	n = 12	HFD (25% fat)	Increased hepatic steatosis Increase in epididymal fat and body fat mass Increased liver TG Increased serum LDL-C No change in serum TG and HDL-C Increases blood glucose Decreased liver glycogen Decreased insulin and insulin/glucagon ratio No change in serum glucagon	↑	↑	Wang et al. (2023)

*BAs* bile acids, *BAT* brown adipose tissue, *eWAT* epididymal white adipose tissue, *FFAs* free fatty acids, *Hba1c* glycated hemoglobin, *HOMA-IR* homeostatic model assessment for insulin resistance, *HDL-C* high-density lipoprotein cholesterol, *HFHC* high-fat/high cholesterol diet, *HFD/HF* high-fat/high-fructose diet, *iWAT* inguinal white adipose tissue, *LDL-C* low-density lipoprotein cholesterol, *eWAT* epididymal white adipose tissue, *mWAT* mesenteric adipose tissue, *pWAT* perirenal white adipose tissue, *rWAT* retroperitoneal white adipose tissue, *SD* Sprague Dawley, *TG* triglycerides, *TC* total cholesterol, *WAT* white adipose tissue

Findings: ↑ increased, improved or higher; ↓ decreased, reduced or lower; ↔ no change in lipid/glucose metabolism

^a^Standard diet contains 10% fat if not otherwise stated

^b^Fat% not stated; NM, not mentioned

Body weights were commonly (92%) recorded, but organ weights were studied in only in 38% of studies (Table 3). Liver steatosis was the most commonly studied parameter (50% of studies), followed by adipose tissue measurements (29% of studies) (Table 3). Blood parameters related to lipid metabolism were studied in 23 studies, and glucose parameters in 14 studies (Table 4). Most common lipid metabolism parameters were measurement of triglycerides (TGs) from either blood or liver (96% of studies), and total cholesterol (TC) in 88% of studies. High-density lipoprotein and low-density lipoprotein cholesterols (HDL-C and LDL-C, respectively) were studied in 50% of studies. Glucose and/or insulin levels as well as glucose and/or insulin tolerances were studied in 58% and 21% of studies, respectively.

**Table 3:** Body weight gain, relative weights of liver and adipose tissue, lipid accumulation

Parameter	Number of studies /all studies	Percentage of studies (%)
Body weight gain	22/24	92
Organ weights (e.g. liver or adipose tissue)	9/24	38
Liver steatosis	12/24	50
Adipose tissue parameters (e.g. adipocyte hypertrophy or lipid accumulation)	7/24	29

**Table 4:** Reported blood parameters related to lipid and glucose metabolism in vivo studies

Biochemical factor	Number of studies/all studies	Percentage of studies (%)
Triglycerides, blood or liver	23/24	96
Total cholesterol	21/24	88
High density lipoprotein cholesterol	12/24	50
Low density lipoprotein cholesterol	12/24	50
Glucose or insulin levels	14/24	58
Glucose or insulin tolerance	5/24	21

Enzymes related to metabolic pathways were commonly studied parameters (42% of studies) (Table 5). Inflammatory markers were studied in 21% of studies. Nuclear receptor activation or expression was studied in 17% of studies. Adipokines and activity of 11β -hydroxysteroid dehydrogenase (11β-HSD1) were both studied in 13% of studies. In few studies also additional markers were analyzed including creatinine, glucose transporter type 4 (GLUT4), and RAR-related orphan receptor gamma (RORγ). Various molecular mechanisms for mycotoxin induced metabolic disruption were proposed including NR activation and mitochondrial effects.

**Table 5:** Main biomarkers and molecular factors in the in vivo studies

Biomarker/molecular factor	Number of studies/all studies	Percentage of studies (%)
Liver injury biomarkers (ALP, ALT, ASP)	11/24	46
Enzymes related to metabolic processes (e.g. 11β-HSD1/2, ACC, CPT1A, FAS, FBP1, HMGCR, HMGCS1, FDFT1, MVD, MVK, SCD1, SQLE, SOD)	10/24	42
Inflammatory factors (e.g. COX-2, CRP, IL-1β, IL-6, TNF-α)	5/24	21
Nuclear receptors (e.g. FABP4, LXR, PPAR-α, PPAR-γ, RORγ)	5/24	21
Protein kinases (e.g. AKT, AMPK, CaMKK, mTOR, PI3K, p70S6K)	4/24	17
Regulators of lipid metabolism (e.g. FOXO3, MYC, SREBP1, SREBP2)	4/24	17
Adipokines (e.g. adiponectin, leptin)	2/24	8
Receptors (e.g. ADIPOR2, CD36, INSR, LDLR)	2/24	8
Adipocyte differentiation markers (e.g. ADRP)	1/24	4
Transporters (e.g. ABCA1, ABCG1, GLUT4)	2/24	8
Intermediates in metabolic pathways (e.g. MDA)	2/24	8
Insulin signaling factors (e.g. IGF-1, IGFALS)	1/24	4

*ABCA1* ATP binding cassette transporter A1, *ABCG1* ATP binding cassette sub-family G member 1, *ACC* acetyl-CoA carboxylase, *ADIPOR2* adiponectin receptor 2, *ADRP* adipose differentiation-related protein, *AKT* protein kinase B, *ALP* alkaline phosphatase, *ALT* alanine aminotransferase, *AMPK* adenosine monophosphate-activated protein kinase, *AST* aspartate aminotransaminase, *CaMKK* calmodulin-dependent protein kinase kinase, *CD36* fatty acid translocase, *C/EBPα* CCAAT/enhancer-binding protein alpha, *COX-2* cyclooxygenase 2, *CPT1A* carnitine palmitoyl transferase alpha, *CRP* C-reactive protein, *FABP4* fatty acid-binding protein 4, *FAS* fatty acid synthase, *FBP1* fructose-1,6-bisphosphatase, *FDFT1* farnesyl-diphosphate farnesyltransferase 1, *FOXO3* forkhead box O3, *GLUT4* glucose transporter 4, *HMGCR* HMG-CoA reductase, *HMGCS1* 3-hydroxy-3-methylglutaryl-CoA synthase 1, *HSD1/2* hydroxysteroid dehydrogenase type 1/2, *IGFALS* IGF acid labile subunit, *IGF-1* insulin growth factor 1, *IL* interleukin, *INSR* insulin receptor, *IRS* insulin receptor substrate, *LDLR* low-density lipoprotein receptor, *LXR* liver X receptor, *MDA* malondialdehyde, *MVD* mevalonate diphosphate decarboxylase, *MVK* mevalonate kinase, *mTOR* mammalian target of rapamycin, *Myc* proto oncogene, *p70S6K* 70-kDa ribosomal protein S6 kinase, *PI3K* phosphoinositide 3-kinase, *PPAR* peroxisome proliferator-activated receptor, *RORγ* RAR-related orphan receptor gamma, *SCD1* stearoyl CoA desaturase, *SOD* superoxide dismutase, *SREBP1* sterol regulatory element binding protein, *SQLE* squalene epoxidase, *TNF-α* tumor necrosis factor alpha, *TREM2* trigger receptor expressed on myeloid cells 2

**Fig. 2:**
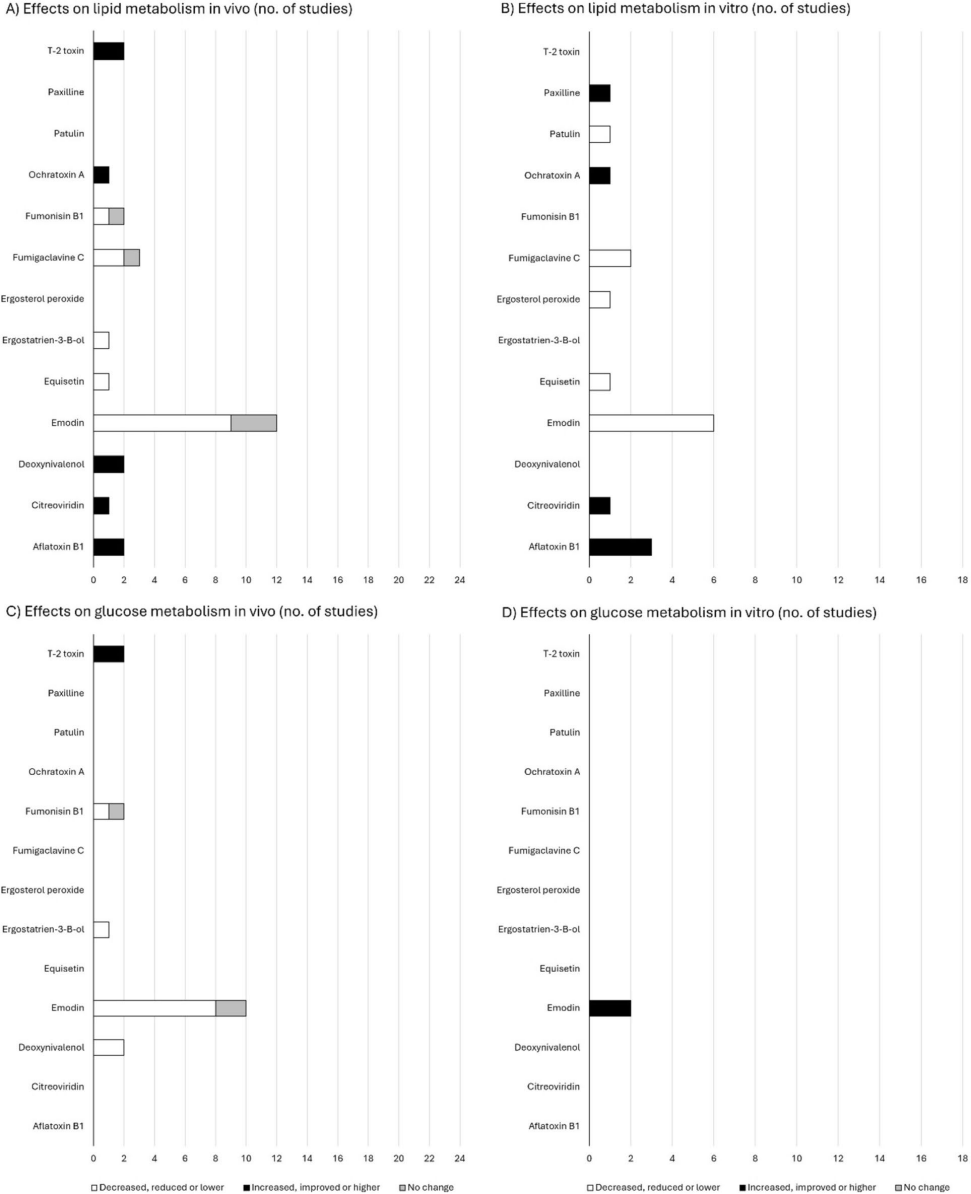
Summary of effects on lipid and glucose metabolism in vivo (**A**, **C**) and in vitro (**B**, **D**) in the included studies for all studied mycotoxins

Based on 24 in vivo studies on mycotoxins and lipid metabolism, 14 reported a decrease in lipid levels or lipid related parameters (Fig. 2). The effect of mycotoxins on metabolic disruption was affected by the type of diet fed. With obesogenic diet, a decrease in lipid metabolism was seen in 14 studies (Xue et al. 2010; Feng et al. 2010; Alisi et al. 2012; Tzeng et al. 2012; Du et al. 2014; Kuo et al. 2015; Li et al. 2016; Wang et al. 2017; Yu et al. 2021, 2023a; Shen et al. 2021; Fang et al. 2022; Xu et al. 2022; Dopavogui et al. 2023), whereas an increase was seen only in one study (Wang et al. 2023). With standard diet, an increase in lipid parameters was noted in two studies (Barbouche et al. 2020; Zong et al. 2022), whereas no change in lipid parameters were seen in three studies (Alisi et al. 2012; Yu et al. 2021; Dopavogui et al. 2023). Five studies did not report composition of the diet (Wang et al. 2012; Feng et al. 2017; Kang et al. 2020; Ren et al. 2020; Che et al. 2023). The positive effects on lipid metabolism were demonstrated by e.g. decrease in triglycerides or total cholesterol (Tzeng et al. 2012; Du et al. 2014), decreased lipid accumulation to liver or adipose tissue (Li et al. 2016; Xu et al. 2022). Some mechanisms were investigated to be involved for example decreased 11β-HSD1 (Feng et al. 2010; Wang et al. 2012; Xu et al. 2022) or increased PPAR-α and farnesoid X receptor (FXR) protein levels (Shen et al. 2021), or decreased PPAR-γ and increase in uncoupling protein 1 (UCP1) levels (Xu et al. 2022).

Emodin was the most common mycotoxin studied, and in all nine studies with obesogenic diet emodin improved lipid metabolism (Xue et al. 2010; Feng et al. 2010; Alisi et al. 2012; Tzeng et al. 2012; Li et al. 2016; Wang et al. 2017; Yu et al. 2021; Shen et al. 2021; Fang et al. 2022), whereas no change in lipid or glucose metabolism was seen with standard diet (Alisi et al. 2012; Yu et al. 2021). Similarly, equisetin (Xu et al. 2022), ergostatrien-3-B-ol (Kuo et al. 2015), fumigaclavine C (Du et al. 2014; Yu et al. 2023b) and fumonisin B1 (Dopavogui et al. 2023) improved lipid metabolism parameters with obesogenic diet whereas Wang et al. 2023 reported opposite results with T2 toxin (Wang et al. 2023). Instead, fumonisin B1 (Dopavogui et al. 2023) had no effect and deoxynivanol (Barbouche et al. 2020; Zong et al. 2022), ochratoxin A (Zheng et al. 2021) worsened the lipid parameters with standard diet. The studies with Aflatoxin B1 (Ren et al. 2020; Che et al. 2023), and citreoviridin (Feng et al. 2017) and T2 toxin (Kang et al. 2020) did not report details of the diet.

The effect of mycotoxins on glucose metabolism was investigated in 17 studies in vivo. With obesogenic diet, different mycotoxins improved glucose metabolism in majority of studies (Xue et al. 2010; Feng et al. 2010; Kobayashi-Hattori et al. 2011; Alisi et al. 2012; Tzeng et al. 2012; Wang et al. 2012; Kuo et al. 2015; Li et al. 2016; Barbouche et al. 2020; Yu et al. 2021; Shen et al. 2021; Dopavogui et al. 2023) and impaired glucose homeostasis in only one study (Wang et al. 2023).With standard diet, improvement of glucose metabolism was reported in one study (Barbouche et al. 2020) but overall mycotoxins did not have effect on glucose metabolism (Alisi et al. 2012; Yu et al. 2021; Dopavogui et al. 2023). Both improvement (Wang et al. 2012) and worsening (Kang et al. 2020) of glucose metabolism was reported in studies where diet was not specified. Emodin (Xue et al. 2010; Feng et al. 2010; Alisi et al. 2012; Tzeng et al. 2012; Li et al. 2016; Yu et al. 2021; Shen et al. 2021), ergostatrien-3-B-ol (Kuo et al. 2015) and fumonisin B1 (Dopavogui et al. 2023) improved both lipid and glucose metabolism and T2 worsened the both. Opposite results were reported with deoxynivalenol, which increased liver steatosis and increased plasma triglyceride levels but decreased insulin fasting blood glucose in mice treated with standard diet (Barbouche et al. 2020).

### Metabolic effects of mycotoxins in vitro

In total 16 in vitro studies were identified regarding mycotoxin exposure and lipid parameters (Table 6). Most commonly studied mycotoxin in terms of lipid metabolism was emodin in 6/16 studies (38%) followed by aflatoxin B1 in three studies, and paxilline, fumigaclavine C and equisetin in two studies. There was one study each on citreoviridin, patulin, ochratoxin A and ergosterol peroxide. In addition, the effect of emodin on glucose metabolism was studied in two studies (Yang et al. 2007; Wang et al. 2012).

**Table 6:** In vitro studies on mycotoxins exposure and lipid metabolism

Mycotoxin	Cell model	Exposure time	Concentration	Induction	Outcome	Lipid	References
Aflatoxin B1	Primary hepatocytes (rat)	0.5–2.5 h	0.1–10 µM	PA	Accumulation of intracellular TG, decreased extracellular TG leakage, no change in conversion of palmitate to TGs	**↑**	Blaude et al. (1992)
	HepG2 HepaRG	48 h	1.25 µM	PA	Increase in mitochondrial lipid droplet size and area	**↑**	Che et al. (2023)
	HepG2 HepG2 Cas9-PTGS2	48 h	5 µM	–	Increased TG and TC, increased AST and ALT	**↑**	Ren et al. (2020)
Citreoviridin	HepG2	24 h	1.25–5 µM	–	Increased lipid accumulation	**↑**	Feng et al. (2017)
Emodin	3T3-L1	48 h	1–50 µM	–	Decreased lipid accumulation and TG	**↓**	Fang et al. (2022)
	THP1	18 h 4–24 h	1–10 µM 1–10 µM	ox-LDL	Increased cholesterol efflux from macrophages	**↓**	Fu et al. (2014)
	Primary hepatocytes (mouse)	24–48 h	12.5–50 µM	OA, PA	Decrease in TG, no change in TC	**↓**	Shen et al. (2021)
	3T3-L1	8 h	0.001–1 µM	–	Decrease in TG content	**↓**	Tzeng et al (2012)
	Primary hepatocytes (rat)	24 h	10–40 µM	FFAs	Reduced accumulation of TG and TC	**↓**	Wang et al. (2017)
	HepG2	24 h	20–80 µM	FFAs	Reduced accumulation of lipids, TG and TC	**↓**	Wang et al. (2017)
Equisetin	3T3-L1 3T3-11β-HSD1-KD	24 h	0.1–10 µM	–	Reduced lipid content	**↓**	Xu et al. (2022)
Ergosterol peroxide	3T3-L1	48 h	10–20 µM	–	Reduced lipid accumulation	**↓**	Jeong and Park (2020)
Fumigaclavine C	Primary peritoneal macrophages (mouse)	4 weeks	Cells form mice treated with 5–20 mg/kg for 4 weeks)	HF/HC-fed ApoE mice	Reduced lipid droplet staining, reduced cholesterol ester	**↓**	Du et al. (2014)
	AML-12	24 h	5- 20 µM	FFAs	Decreased hepatic TG levels	**↓**	Yu et al. (2023a)
Ochratoxin A	HepG2 Primary hepatocytes (mouse)	24 h 6–48 h	5–15 µM 10 µM	–	Increase in lipid droplet deposition and TGs	**↑**	Zheng et al. (2021)
Patulin	AML12 HepG2	24–48 h	0.1–1 µM	OA, PA	Decreased lipid accumulation	**↓**	Yu et al. (2023b)
Paxilline	Primary hepatocytes (human) HepG2	24.5 h	1–50 µM	OA, PA	Increased lipid accumulation	**↑**	Moya et al. (2010)

*3T3-L1* mouse preadipocyte (differentiated), *3T3-11β-HSD1-KD* mouse preadipocyte (modified, 11β- hydroxysteroid dehydrogenase type 1 knock-down), *ALT* alanine aminotransferase, *AML12* mouse hepatocyte, *AST* aspartate transaminase, *FFA* free fatty acids, *HepG2* human hepatocellular carcinoma cell line, *HepaRG* human hepatocellular carcinoma cell line (differentiated); HepG2 Cas9-PTGS2 (modified, cyclooxygenase-2 knockdown HepG2 cell line), *HFD/HF* high-fat/high-fructose diet, *OA* oleic acid, *ox-LDL* oxidized low-density lipoprotein, *PA* palmitic acid, *RAW* *264.7* mouse macrophages, *TG* triglycerides, *TC* total cholesterol, *THP1* human macrophages (differentiated)

Findings: ↑ increased, improved or higher; ↓ decreased, reduced or lower

Most commonly used cell line was HepG2 (human hepatoma) cell line (7/18 studies; 39%) followed by 3T3-L1 (mouse inducible adipocytes) in 6/18 studies (33%). Mouse hepatocyte AML-12 cell line, and human monocytic leukemia THP-1 cells were used in two studies, and human hepatocytes, rat hepatocytes, mouse peritoneal macrophages, isolated mouse hepatocytes, cyclooxygenase-2 knockdown cell line and human kidney HEK-293 T cells were used in one study. The exposure times varied from 6 to 48 h with the most common exposure time was 24 h. The test concentrations varied between 0.001 µM and 80 µM.

All studies made in liver cells studied lipid or cholesterol accumulation (Moya et al. 2010; Wang et al. 2017; Feng et al. 2017; Ren et al. 2020; Shen et al. 2021; Zheng et al. 2021; Xu et al. 2022; Che et al. 2023; Yu et al. 2023a, b). Studies performed with 3T3-L1 cell line assessed lipid accumulation (5 out of 6, 83%) or glucose uptake, and the role of insulin in glucose uptake (Wang et al. 2012; Yang et al. 2023). Glucose uptake was not studied in liver cells.

Several signalling pathways and NRs were reported to take part to lipid accumulation, such as Rho associated protein kinase-Ras homolog family member A pathway (Rock-Rhoa) (Yu et al. 2023b) and c-Jun N-terminal kinase (JNK)—extracellular-signal regulated kinase (ERK) pathways (Jeong and Park 2020; Fang et al. 2022) as well as PPAR-γ (Fu et al. 2014; Jeong and Park 2020; Zheng et al. 2021; Fang et al. 2022; Xu et al. 2022; Che et al. 2023) and SREBP1 transcription factors (Wang et al. 2017; Che et al. 2023; Yu et al. 2023a, b) (Table 7).

**Table 7:** Main biomarkers and molecular factors in the in vitro studies on lipid and glucose metabolism

Biomarker/molecular factor	Number of studies/all studies	Percentage of studies (%)
Nuclear receptors (e.g. LXR-α, PPAR-α, PPAR-γ, RORγ)	9/18	50
Regulators of lipid metabolism (e.g. C/EBPα, FABP4, SREBP1)	7/18	39
Enzymes related to metabolic processes (e.g. 11β-HSD1, ACC, ATGL, CPT1A, FAS, HSL, SCD1)	7/18	39
Protein kinases (e.g. AMPK, CaMKK, ERK, JNK, mTOR, p38, ROCK, RhoA, p70S6K)	4/18	22
Intermediates in metabolic pathways (e.g. FPP, GGPP)	1/18	6
Inflammatory factors (e.g. IL-1β, IL-6, TNF-α)	2/18	11
Transporters (e.g. ABCA1, ABCG1, UCP1)	1/18	6

*ABCA1* ATP binding cassette subfamily A member 1, *ABCG1* ATP binding cassette sub-family G member, *ACC* acetyl-CoA carboxylase, *ATGL* adipose triglyceride lipase, *AMPK* adenosine monophosphate-activated protein kinase, *C/EBPα* CCAAT/enhancer-binding protein alpha, *CPT1A* carnitine palmitoyl transferase alpha, *ERK* extracellular-signal regulated kinase, *FAS* fatty acid synthase, *FABP4* fatty acid-binding protein 4, *FPP* farnesyl diphosphonate, *GGPP* geranylgeranyl diphosphonate, *HSD1* hydroxysteroid dehydrogenase type 1, *HSL* hormone sensitive lipase, *IL* interleukin, *JNK* c-Jun N-terminal kinase, *LXR-α* liver X receptor alpha, *mTOR* mammalian target of rapamycin, *p38* p38 mitogen-activated protein kinase, *p70S6K* 70-kDa ribosomal protein S6 kinase, *PPAR* peroxisome proliferator-activated receptor, *ROCK* Rho associated protein kinase, *RhoA* Ras homolog family member A, *SCD1* stearoyl CoA desaturase, *SREBP1* sterol regulatory element binding protein 1, *TNF-α* tumor necrosis factor alpha, *UCP1* uncoupling protein 1

Changes in PPAR-γ expression and activity after mycotoxin treatment were observed in several studies, e.g., increased mRNA and protein expression by emodin in ox-LDL stimulated THP1 macrophages (Fu et al. 2014), activation by ochratoxin A and aflatoxin B1 in HepG2 cells (Zheng et al. 2021; Che et al. 2023) and repression by equisetin and ergosterol peroxide in 3T3-L1 cells (Jeong and Park 2020; Xu et al. 2022). Emodin treatment was also shown to increase liver X receptor (LXR) α mRNA and protein expression in ox-LDL stimulated THP1 macrophages (Fu et al. 2014), while the known LXRα agonist paxilline increased lipid accumulation in HepG2 cells and human hepatocytes (Moya et al. 2010).

The results of in vitro studies (Table 6) are in line with those of in vivo studies (Table 2). The majority of the studies observed that mycotoxins reduce lipid accumulation established by fat induction in liver and adipose cell lines, but also opposite findings were reported. Similarly to in vivo studies, emodin was most studied mycotoxin showing improvement of lipid metabolism and emodin also improved glucose metabolism in two studies (Yang et al. 2007; Wang et al. 2012). On the other hand, aflatoxin B1, citreoviridin, ochratoxin A and paxilline (Blaude et al. 1992; Moya et al. 2010; Ren et al. 2020; Zheng et al. 2021; Che et al. 2023) impaired lipid metabolism.

Emodin was the only mycotoxin studied regarding glucose metabolism in vitro. In a study of Wang et al. (2012), incubation with 3 μmol/L of emodin significantly reversed the impaired insulin-stimulated glucose uptake induced by 11-dehydrocortisone (11-DHC), although emodin treatment alone caused reduction in insulin-stimulated glucose uptake in 3T3-L1 cells (Wang et al. 2012). In addition, emodin was shown to increase basal and insulin-stimulated glucose-uptake in differentiated 3T3-L1 adipocytes (Yang et al. 2007).

## Discussion

Our systematic literature search with the input of 76 mycotoxins as keywords yielded in total studies on 13 different mycotoxins. In total 31% of the studies focused on emodin, but despite a low number of publications on other mycotoxins, it can be concluded that mycotoxins do not have a uniform effect on metabolism, but individual mycotoxins differ in their metabolic disruption potential. For example, aflatoxin B1 exposure indicated impairment of lipid metabolism (Ren et al. 2020; Zheng et al. 2021; Che et al. 2023), whereas fumigaclavine C was found to improve metabolic parameters (Du et al. 2014; Yu et al. 2023b). Moreover, mycotoxin exposure alone was not sufficient enough to have an effect on metabolism, but in combination with HFD, mycotoxins often improved lipid metabolism.

Various mechanisms were proposed for emodin—modulation of cytokine and MAPK pathways (Fang et al. 2022), decreasing secretion of inflammatory markers from adipose tissue by changing macrophage polarization to anti-inflammatory phenotype (Yu et al. 2021), and inhibition of glucocorticoid activating enzyme, leading to reduction of adverse excess glucocorticoid effects, such as increased adipose tissue accumulation or decreased insulin sensitivity (Feng et al. 2010). For other mycotoxins, ergostatrien-3-B-ol exposure regulated fatty acid oxidation and lipogenesis related genes (PPAR-α and diacylglycerol O-acyltransferase 2) contributing to decreased triglycerides in blood, and increased Akt pathway activation leading to increased insulin sensitivity by improving GLUT4 translocation (Kuo et al. 2015). Fumigaclavine C inhibited hepatic de novo lipogenesis and proposedly contributed to reduced liver steatosis by decreasing prenylated proteins (geranylgeranyl diphosphate GGPP and farnesyl diphosphate FPP) (Yu et al. 2023b). Also, high FB1 exposure led to improvement in certain metabolic health parameters, such as hepatic lipid content and blood glucose, while adverse effects (e.g. increase in liver inflammation) were also observed. Gut microbiota effects (demonstrated by e.g. changes in α-diversity) were restored by FB1 treatment during HFD, but still gut dysbiosis was introduced. Altered sphingolipid metabolism was presented after HFD combined with FB1 exposure, that affects cell homeostasis presumably by disruption of endolysosomal trafficking pathway (Dopavogui et al. 2023). Thus, the published studies suggest that emodin has potential to reduce already established lipid accumulation, although more evidence is needed to draw the final conclusions.

Triglycerides and cholesterol were the most commonly studied parameters both in in vitro and in vivo studies, followed by cholesterol transporter measurements in vivo (HDL-C or LDL-C). The effect of mycotoxins on glucose metabolism was not studied as extensively as on lipid metabolism. Both in vivo and in vitro studies had the same endpoints such as lipid accumulation to hepatocytes or adipocytes, although in vitro studies usually aimed to determine underlying molecular mechanism as well. Especially NRs and other lipid metabolism related proteins (e.g. UCP1 or lipid conversion enzymes) were commonly studied in in vitro*.*

In addition to common lipid or glucose metabolism parameters, additional pathways and mediators of metabolic disruption were investigated. Effects of mycotoxins on microbiota was studied by (Dopavogui et al. 2023) where they found fumonisin B1 restoring microbiota alpha diversity disturbed by high fat diet. It seems that disturbances to microbiota alpha diversity are linked with obesity in children (Alcazar et al. 2022). Some proposed mechanisms for exerting negative effects to metabolic health were activation perilipin 2 (PLIN2) pathway involving e.g. Microtubule Associated Protein 1 Light Chain 3 Beta (LC3B), protein 53 (p53) and protein 62 (p62) proteins (with decreased LC3B) (Che et al. 2023), disruption of pathway involving PTEN induced kinase 1 (PINK1), ubiquitin E3 ligase (Parkin), and LC3B (Ren et al. 2020), and increased RORγ activation (Zong et al. 2022).

Thus, it seems that different mycotoxins do not share the pathways in regard to metabolic health, although some similarities can be identified, such as increase in lipid metabolism regulator PPARγ expression by different mycotoxins (Fang et al. 2022; Che et al. 2023), however, levels of PPAR-α isoform were found to be reduced by emodin exposure (Shen et al. 2021). As some NRs may have various roles in regards to metabolic health in different tissues (Ahmadian et al. 2013; Lv et al. 2022), conflicting findings can be expected, as is the case of activation of LXR where its activation increased cholesterol efflux in macrophages (Fu et al. 2014), but reduced accumulation of lipids in hepatocytes (Moya et al. 2010).

Contrary to our hypothesis, these results suggest a direction towards better metabolic health instead of metabolic disruption, although the link is mostly representative only for single mycotoxin emodin. However, mycotoxins can cause a variety of adverse health effects, including cancer (Schrenk et al. 2020a), endocrine disruption (Alexander et al. 2011), and immunosuppression (Knutsen et al. 2017)), thus it is not recommended to use mycotoxins to improve metabolic health. In addition, emodin is prohibited in foods in European union (EC 1925/2006). However, as some mycotoxins have potential for reducing e.g. triglyceride levels, the structures could be useful in identifying new drug candidates for metabolic diseases.

### Limitations of the study

Metabolic disruption as a wide term including main metabolic organs (adipose tissue, liver, pancreas), and many kind of effects besides general clinical signs of metabolic syndrome, such as neuroendocrine control of appetite related hormones or thyroid disruption (Heindel et al. 2022). Thus, apart from the findings of our study, more evidence of the “secondary” effects to metabolic disruption should be gathered in order to make reliable estimation of the metabolic disruption potential of mycotoxins. For example, our study excluded studies on how mycotoxins affect preadipocyte differentiation, although that may be an important part of metabolic disruption (Heindel et al. 2022). As detailed quality check of the studies (e.g. purity of compounds, suitable carriers, feed or water intake, diet quality of animals) was not included in this review, a level of uncertainty is present in the results. In addition, selected studies often report only associations between mycotoxins exposure to certain, well-defined endpoints, such as lipid accumulation to hepatocytes or adipocytes, but causality was not established. In addition, it is difficult to draw conclusions due to the wide variety in the focuses of the studies and the molecular factors analyzed.

The doses used in the studies are considered high considering clinical relevance (Table 2). For example, in risk assessment by EFSA (Schrenk et al. 2020a), the highest 95th percentile exposure group in Europe is exposed to aflatoxin B1 for 14.01 ng/kg bw/day, that is more than 1000-fold below the lowest amount of aflatoxin B1 exposure identified from (Ren et al. 2020) study (0.6 mg/kg bw/day). In addition, exposure to deoxynivalenol is well below the doses identified in this systematic literature search—EFSA identified a highest 95th percentile to be exposed to deoxynivalenol for 3.7 µg/kg bw/day (Knutsen et al. 2017), when in our systematic literature search the lowest exposure dose in vivo was 4 mg/kg in pigs (Zong et al. 2022). However, since these doses seem to cause adverse effects, they can be identified as lowest observed adverse effects levels, but a no observed adverse effect level cannot be identified, although in some studies the lowest exposure dose was not causing significant effects.

## Conclusions

As demonstrated by the evaluated in vivo and in vitro studies, mycotoxins seem to have more positive than negative effects on metabolism, however, there were differences between mycotoxins and this finding cannot be generalized to mycotoxins as a group. In addition, different regulatory pathways behind the metabolic effects were suggested, although some similarities between studies were observed. Based on the available data, a general conclusion on the role of mycotoxins as a group acting as metabolic disruptors cannot be made. Characterization of the different mediators of potential adverse metabolic effects of mycotoxins is needed to distinguish the mechanisms of action of different mycotoxins. As mycotoxin exposure in some cases indicated improved metabolic health in, they may have further potential to aid in drug discovery and development.

## Data Availability

Data sharing not applicable to this manuscript as no datasets were created or analyzed during the current study.
